# Identification of Anti‐Inflammatory and Analgesic Mechanisms of Naru‐3 Wei Pill Using Animal Models, UHPLC‐QE‐MS, and Integrated Network Pharmacology

**DOI:** 10.1155/mi/5579410

**Published:** 2026-04-29

**Authors:** Zhiqiang Han, Husile Kang, Aidi Zhang, Qigeqin, Baolechaolu, Wulanqiqige, Huan Wang, Yanhua Xu, HaSi, Anda, Lan Xue

**Affiliations:** ^1^ Institute of Clinical Pharmacology of Traditional Mongolian Medicine, Affiliated Hospital of Inner Mongolia Minzu University, No.1742, Huolinhe Street, Horqin Area, Tongliao, 028000, Inner Mongolia, China, nmgmzdxfsyy.com; ^2^ Department of Medical Education, Affiliated Hospital of Inner Mongolia Minzu University, Tongliao, 028000, Inner Mongolia, China, nmgmzdxfsyy.com; ^3^ Clinical Medical College, Inner Mongolia Minzu University, Tongliao, 028000, Inner Mongolia, China, imun.edu.cn; ^4^ Medical Innovation Center for Nationalities, Inner Mongolia Medical University, Huhhot, 010110, Inner Mongolia, China, immu.edu.cn; ^5^ College of Mongolian Medicine, Inner Mongolia Medical University, Huhhot, 010110, Inner Mongolia, China, immu.edu.cn

**Keywords:** analgesic, anti-inflammatory, blood-borne components, molecular dynamics, Naru-3 Wei Pill, network pharmacology

## Abstract

**Background:**

Naru‐3 Wei Pill is a traditional Mongolian Medicine (TMM) that has anti‐inflammatory, analgesic, and antibacterial effects. The present study aimed to demonstrate the anti‐inflammatory and analgesic effect of Naru‐3 Wei Pill, identify the core components and key targets and determine the pharmacological basis and mechanism of its blood‐borne components.

**Methods:**

The anti‐inflammatory and analgesic effects of Naru‐3 pills were studied using hot plate, tail flick, and acetic acid–induced writhing tests. The blood‐borne components of Naru‐3 Wei Pill were identified using ultrahigh‐performance liquid chromatography with Q‐Exactive mass spectrometry (UHPLC‐QE‐MS), and their targets were screened using ChEMBL, TCMIO, GeneCards, and TTD databases. Network construction, Gene Ontology (GO) and Kyoto Encyclopedia of Genes and Genomes (KEGG) analysis, and protein–protein interaction (PPI) network was performed. Molecular dynamics methods, PCR and western blot, were used for verification of the results.

**Results:**

Multiple pain models results indicate that Naru‐3 pills exert anti‐inflammatory and analgesic effects by modulating the PI3K signaling pathways and reducing inflammation factors (interleukin [IL]‐6). A total of 35 blood‐borne components were identified, acting on 291 targets involving 172 pathways. The core targets included AKT1, SRC, mitogen‐activated protein kinase 14 (MAPK14), and ESR1. Molecular docking and dynamics simulations conformed strong binding affinity of the complexes formed between Rhein and AKT1, MAPK1, and SRC, and between genistein and SRC. PCR and western blot confirm the regulation of AKT/PI3K pathway of Naru‐3 pills in animal models.

**Conclusion:**

Naru‐3 pills significantly prolonged pain thresholds and reduced pain behaviors in mice. The study identified the material basis and mechanisms of its anti‐inflammatory and analgesic effects, providing a foundation for further research.

## 1. Introduction

Naru‐3 Wei Pill is a common Mongolian medicine that is described in the classic work “Supreme Formula” (Zhi Gao Yao Fang) [1] and was included in the “Drug Standards of the Ministry of Health: Mongolian Medicine” in 1998 [2]. This prescription is formulated as a red water pill or paste pill and is composed of three Chinese herbs: *Terminalia chebula Retz* (He Zi), *Aconitum kusnezoffii Reichb*. (Zhi Cao Wu), and *Piper longum L*. (Bi Ba). It has a slightly sour taste and a pungent and mild nature, and its functions include dispelling yellow water and Badagan Heyi, eliminating stickiness, and relieving pain. It is mainly used to treat in arthralgia yellow water disease, insect disease, diphtheria, chickenpox and other viscous diseases, and waist and crotch pain [2]. Pharmacological studies have shown that Naru‐3 can prevent inflammation and neovascularization and alleviate rheumatoid arthritis in collagen‐induced arthritis in rats [3]. Naru‐3 also exerts antiapoptotic effects in intervertebral disc degeneration through delaying extracellular matrix degradation and accelerating cell proliferation [4], has an impact on inflammatory cytokine expression, and can be used to effectively treat traumatic spinal cord injury in rats [5]. The analgesic effects of Naru‐3 on neuropathic pain may be associated with negative regulation of the p38/interleukin (IL)‐1β inflammatory loop, and mediated by the MMP9/IL‐1β signaling pathway, during activation of microglia [6]. However, studies of the mechanisms of action of Naru‐3 Wei Pill have mostly considered its effects on individual targets, meaning that we lack a comprehensive understanding of its anti‐inflammatory effects and characteristics.

Network pharmacology is widely used to study the relationships between drugs and diseases. It integrates experimental and clinical investigations with data processing methods, providing support for clarification of drug mechanisms and development of new drugs [7]. Here, we construct a multi‐level drug–target–disease network, consistent with the holistic and systematic perspective of Mongolian medicine and reflecting the effects of Naru‐3 on multiple compounds, targets, and pathways. Such an approach enables a thorough elucidation of the mechanisms underlying the various effects of drugs, providing insights into the efficacy of Traditional Chinese Medicine and its compounds [8].

In the present study, we used ultra‐high performance liquid chromatography Q‐Exactive mass spectrometry (UHPLC‐QE‐MS) technology to analyze the anti‐inflammatory and analgesic targets of Naru‐3 Wei Pill in mice after intragastric administration. Blood‐borne components of the prescription were identified, and their anti‐inflammatory and analgesic targets and pathways were studied through network pharmacology. The results of this analysis were combined with computational pharmaceutical validation to elucidate the mechanisms of the anti‐inflammatory and analgesic effects of Naru‐3 Wei Pill and to provide a basis for the screening of quality markers.

## 2. Materials and Methods

### 2.1. Animal Experiments With Mice

For acetic acid–induced writhing analgesic model, a total of 60 SPF‐grade ICR mice, half male and half female, with body weights ranging from 18 to 20 g, were randomly divided into six groups by body weight: blank group, model group, positive control group, and Naru‐3 Pill low‐, medium‐, and high‐dose groups, with 8 mice in each group. The blank group and model group were given distilled water by gavage; the positive control group was administered aspirin suspension at a dose of 0.36 g/kg by gavage; the Naru‐3 Pill low‐, medium‐, and high‐dose groups were given the drug at doses of 0.2, 0.4, and 0.8 g/kg Naru‐3 Wei Pill suspension (manufactured by Kulun Mongolian Medicine Factory), respectively, by gavage. The gavage volume was uniformly 20 mL/kg for all groups, and gavage was performed once every 4 h for a total of two consecutive administrations. Mice were anesthetized and sacrificed by intraperitoneal injection of an excessive dose of pentobarbital sodium solution (150 mg/kg).

For Naru‐3 Wei Pill, based on the required final concentration of the suspension for the experiment, accurately weigh a specified amount of Naru‐3 Pill fine powder. Add the powder slowly in portions to the 0.5% (*w*/*v*) sodium carboxymethylcellulose (CMC‐Na) suspending agent, stirring continuously while adding, until a homogeneous suspension with no obvious particle sedimentation is formed. Transfer the well‐mixed suspension into a volumetric flask. Rinse the original container with a small amount of 0.5% (*w*/*v*) CMC‐Na solution and combine the rinsings into the volumetric flask. Add the suspending agent to dilute the solution to the required volume. Prepare the suspension immediately before use.

For the tail flick test, the mice were placed in a pain meter, and the time to tail flick was recorded as the baseline thermal pain threshold. The experimental conditions for the tail flick test were set at 22 V, 280 W, and 16 mm, and the mice with a threshold around 7 s were selected for the study.

For the hot plate test, the mice were placed in a preheated YLS‐6B intelligent hot plate apparatus at 55 ± 0.1°C, and the time to lick the hind paw was recorded as the pain threshold. The average of two measurements was used as the baseline hot plate pain threshold. The thermal pain threshold was measured 30 min after the last dose. Blood was collected via retro‐orbital puncture, and PI3K (#57320, cell signaling) levels were measured using enzyme‐linked immunosorbent assays (ELISAs).

For the acetic acid–induced writhing test, 30 min after the last dose, mice were intraperitoneally injected with 0.6% acetic acid (0.2 mL per mouse) to induce pain. The number of writhing movements within 20 min and the time to the first writhing movement were recorded. Blood was collected via orbital puncture, and serum levels of IL‐6 (PI326, Beyotime) and PGE2 (abs554101, Absin) were measured using ELISA.

### 2.2. Animal Experiments With Rats

A total of 60 male Sprague‐Dawley rats with a body weight of 180–220 g were used for rat paw edema inflammation experiment. Rats were acclimated for 1 week in the laboratory environment of the Clinical Pharmacology Laboratory of the Affiliated Hospital of Inner Mongolia University for Nationalities, with a room temperature of 22–24°C and relative humidity of 55%–56%. The rats were randomly divided into six groups: blank group, model group, positive group (aspirin), Naru‐3‐flavor pill low‐, medium‐, and high‐dose groups. The drug doses were as follows: aspirin suspension at 0.19 g/kg for the positive group, 0.105, 0.21, and 0.42 g/kg for the low‐, medium‐, and high‐dose groups. The rats were gavaged with the corresponding drugs for 1 week, with a gavage volume of 20 mL/kg.

On the 7th day, 1 h after gavage, the blank group was injected with 0.9% saline (0.1 mL/rat) to induce modeling, while the other groups were injected with 1% carrageenan suspension (0.1 mL/rat) into the right hind paw to induce inflammation. The paw volume was measured once before modeling and at 1, 2, 3, 4, and 5 h after modeling. The paw edema rate was calculated using the formula: edema rate = [(post‐inflammatory paw volume − pre‐inflammatory right hind paw volume)/pre‐inflammatory volume] × 100%. ELISA method was used to measure the levels of IL‐6 (PI328, Beyotime), IL‐1β (PI303, Beyotime), TNF‐α (Pt516, Beyotime), and PGE2 (CSB‐E07967r, CUSABIO) in the serum with corresponding ELISA kits. The paw tissues were collected for RT‐qPCR and western blot detection of AKT, PI3K, and mTOR expression.

### 2.3. Preparation of Loading Samples

Eighteen SPF‐ ICR mice (18–22 g) were randomly divided into three groups (blank, 0.5 h, and 1 h), with six mice in each group. Naru‐3 Wei Pill suspension (Kulun Mongolian Medicine Factory) was prepared and administrated to mice by gavage at a dose of 16 g kg^−1^, calculated by the body surface area method [5]. The blank group was given the same amount of distilled water. Fasting and water deprivation were performed 24 h before administration. After 0.5 and 1 h of administration, blood was extracted and centrifuged at 4°C for 20 min at 2980 × *g*. Plasma was collected and stored at −80°C until use. All animal experiments were performed in line with the guidelines of the Affiliated Hospital of Inner Mongolia Minzu University (Protocol Number NM‐LL‐2023‐03‐15‐04).

Serum samples (0.4 mL) were collected, and 40 μL of hydrochloric acid (2 mol/L) was added. The mixture was vortexed for 1 min and then allowed to stand at 4°C for 15 min; this process was repeated four times. Then, 1.6 mL of acetonitrile (Q2240576, Technologies) was added, followed by vortexing for 5 min and centrifuging for 5 min. The supernatant was removed (about 1800 μL) and dried with nitrogen. Finally, 150 μL of 80% methanol (10 μg/mL) was added for remixing, and the mixture was centrifuged for 5 min. The supernatant was obtained for testing.

### 2.4. UHPLC‐QE‐MS Analysis

UHPLC detection was conducted using a Vanquish UHPLC system (Thermo Fisher). The chromatographic column was a UPLC BEH C18 (2.1 mm × 100 mm, 1.7 μm; Catalog Number 186002352); the mobile phase was 0.1% formic acid aqueous solution (A), 0.1% formic acid–acetonitrile (B); the injection volume was 5 μL. The MS settings were as follows: electrospray ion source ion mode (+/−); sheath gas velocity: 45 Arb; auxiliary gas velocity: 15 Arb; full MS resolution: 70,000; MS/MS resolution: NCE mode; injection voltage: −3.6 kV. XCMS software was used for data analysis and to perform operations including peak recognition, peak extraction, and peak integration. Using the secondary mass spectrometry database and thermal decomposition matching method, peaks containing MS/MS data were identified.

### 2.5. Screening of Blood‐Based Components of Naru‐3 Wei Pill

The targets of blood‐borne components of Naru‐3 were identified using the ChEMBL database and TCMIO, and metabolites were screened based on their absorption rate and drug‐likeness (DL). The screening criteria were: percentage human oral absorption ≥30% and DL ≥0.18.

### 2.6. Acquisition of Disease Targets

The TTD and GeneCards databases were searched using keywords “Inflammation” and “Pain” to obtain disease targets. These disease targets were intersected with the gene targets of the blood‐borne components.

### 2.7. Establishment of Component‐Target Network

Cytoscape 3.9.1 was used to establish networks based on the interactions of compounds of Naru‐3 Wei Pill and their targets.

### 2.8. Construction of the Protein–Protein Interaction (PPI) Network

Compounds and disease targets were retrieved for cross‐processing, and overlapping targets were identified and imported into the STRING database. After determination of protein relationships, the results were imported into Cytoscape 3.9.1 for visualization and to obtain a PPI network.

### 2.9. Gene Ontology (GO) and Kyoto Encyclopedia of Genes and Genomes (KEGG) Enrichment Analyses

The GO database stores various functional information about genes, including biological processes and molecular functions, which can be used to determine the biological molecular mechanisms of drugs. The KEGG database can also be used to determine biological functions and identify candidate targets. In the present research, the DAVID database (https://david.ncifcrf.gov/) was used for GO and KEGG enrichment analysis of targets, using a threshold <0.05, and the results were visualized through the Weishengxin online platform.

### 2.10. Molecular Docking

Protein and small‐molecule PDB structure files were obtained from the Protein Data Bank and ZINC15, respectively. PyMOL was used to remove water and ligands from protein macromolecules, and Chem3D was used to optimize the structures of small molecules. Proteins and small molecules were then added polar hydrogens using AutoDockTools, and rotatable bonds were assigned for small molecules, with the results saved as pdbqt files. The docking region was set with autogrid, the docking region information file was exported, and the molecules were docked using AutoDock Vina software.

### 2.11. qPCR Analysis

Total RNA of each samples were extracted using Trizol reagent (Invitrogen), and reverse‐transcribed into cDNA with the PrimeScript RT Master Mix kit (Takara Bio, Kusatsu, Japan). Quantitative PCR (q‐PCR) was performed to measure the mRNA expression levels of the hub genes, utilizing SYBR Green PCR Mix (Monad Biotech, Wuhan, China) on a CFX Connect Real‐Time PCR Detection System (Applied BIO‐RAD, Hercules, CA, USA). In this process, β‐actin served as the internal reference gene. The specific primers employed for q‐PCR were as follows: AKT: forward: 5′‐AGGAGGTCATCGTTGCCAAGGA‐3′; reverse: 5′‐CGCTCACGAGACAGGTGGAAGA‐3′; PI3K: forward: 5′‐GACCTGTGCCTTCTGCCTTACG‐3′; reverse: 5′‐GCAATCGTCGTGGCGTCCTT‐3′; mTOR: forward: 5′‐TGTGGCAAGAGCGGCAGACT‐3′; reverse: 5′‐AGGGTGAACTGTTGGCAGAGGA‐3′.

### 2.12. Western Blot Analysis

Protein extraction from liver tissues was conducted using RIPA buffer (Beyotime, Shanghai, China) supplemented with protease inhibitor, phosphatase inhibitor, and Phenylmethylsulfonyl fluoride (PMSF). A total of 30 μg of protein was separated by sodium dodecyl sulfate polyacrylamide gel electrophoresis (SDS‐PAGE) and subsequently transferred onto polyvinylidene fluoride (PVDF) membranes. The membranes were blocked with 5% BSA for 2 h at room temperature, followed by incubation with primary antibodies against AKT (proteintech, 10176‐2‐AP), PI3K (CST, 4228), mTOR (Cell Signaling, 2983S), and Vinculin (ABclonal, A2752) at a 1:1000 dilution in 3% BSA at 4°C overnight. After washing the membranes three times with TBST for 10 min each, they were incubated with secondary antibodies at room temperature for 2 h. The protein bands were visualized using an ECL chemiluminescence kit (Beyotime, Shanghai, China). Vinculin served as the loading control, and densitometric analysis of the bands was performed using ImageJ software.

### 2.13. Statistical Analysis

Statistical analysis were performed using GraphPad Prism 8.3.0 software (San Diego, CA, USA). Experimental data are expressed as the mean ± standard deviation. For comparisons between groups, one‐way analysis of variance was applied, and a *p*‐value of <0.05 as statistical significance.

## 3. Results

### 3.1. The Anti‐Inflammatory and Analgesic Effect of Naru‐3 Pills

We evaluated the anti‐inflammatory and analgesic effects of Naru‐3 pills using the hot plate, tail flick, and acetic acid–induced writhing tests and measured serum levels of PI3K, PGE2, and IL‐6. In the hot plate test, the model group showed significantly higher lick counts (10.88 ± 4.02) and lick durations (16.04 ± 5.50 s) than the control group at 30 min (*p* < 0.05; Table 1). The positive (aspirin) and Naru‐3 pill groups had significantly lower lick counts and durations, with the middle‐dose group showing the longest lick duration (23.09 ± 15.48 s). In the tail flick test, the model group had shortened latencies (5.29 ± 0.93 s) compared to control group (7.14 ± 0.62 s), while all Naru‐3 pill groups exhibited prolonged latencies (*p* < 0.05; Table 1). These findings suggest that Naru‐3 pills may modulate the analgesic effects.

**Table 1:** Analgesic effects of Naru‐3 Wei Pill on mice using hot plate test.

Group	Number of hind paw licks at 30 min	Hind paw lick duration (s) at 30 min	Tail flick latency (s)
Control	—	—	7.14 ± 0.62
Model	10.88 ± 4.02	16.04 ± 5.50	5.29 ± 0.93
Positive	10.63 ± 4.17	14.33 ± 4.13	9.84 ± 1.25^a^
Low‐Naru‐3	9.00 ± 3.21	18.54 ± 6.55	9.38 ± 2.11^a^
Middle‐Naru‐3	7.25 ± 3.92	23.09 ± 15.48	10.33 ± 3.44^a^
High‐Naru‐3	9.25 ± 2.96	17.07 ± 3.79	9.61 ± 3.12^a^
*F*	1.081	1.206	5.863
*p*	0.385	0.323	0.001

^a^indicates a significant difference compared with the model group (*p* < 0.05).

In the acetic acid–induced writhing test, the model group had significantly more writhing movements (47.13 ± 34.49) than the control group (*p* < 0.05; Table 2). The positive and Naru‐3 pill groups had fewer writhing movements, with the high‐dose group showing the lowest count (9.75 ± 5.55). The time to the first writhing movement was also prolonged in these groups (*p* < 0.05). We observed that serum IL‐6 levels were significantly elevated in the model group (221.88 ± 10.43 pg/mL, *p* < 0.05) compared with the control group. Treatment with low‐ and high‐dose Naru‐3 pills significantly reduced IL‐6 levels relative to the model group (*p* < 0.05), whereas the middle‐dose Naru‐3 pill group showed no significant reduction. No significant differences in PGE2 levels were observed among groups (Table 2). These results indicate that Naru‐3 pills exert anti‐inflammatory and analgesic effects by reducing inflammation, as evidenced by decreased IL‐6 levels.

**Table 2:** Analgesic effects of Naru‐3 Wei Pill on mice using acetic acid–induced writhing test.

Group	PGE2 (pg/mL)	IL‐6 (pg/mL)	Number of writhing movements	Time to first writhing (s)
Control	83.96 ± 11.61	195.83 ± 15.60^a^	—	—
Model	98.62 ± 13.89	221.88 ± 10.43^b^	47.13 ± 34.49	283.25 ± 175.57
Positive	83.67 ± 21.03	215.00 ± 16.06^b^	9.00 ± 16.88^a^	736.25 ± 498.00^a^
Low‐Naru‐3	85.25 ± 22.40	213.55 ± 19.32	10.00 ± 11.17^a^	625.88 ± 418.48
Middle‐Naru‐3	81.12 ± 15.55	223.57 ± 14.21^b^	13.63 ± 7.15^a^	245.25 ± 49.28
High Naru‐3	79.81 ± 8.09	207.58 ± 21.49	9.75 ± 5.55^a^	480.50 ± 332.75
*F*	1.396	3.025	6.425	3.180
*p*	0.246	0.020	<0.01	0.025

^a^indicates a significant difference compared with the model group (*p* < 0.05).

^b^indicates a significant difference compared with the control group (*p* < 0.05).

In the rat paw edema inflammation experiment, Table 3 shows the comparison of paw edema rates at different time points among the groups. The model group exhibited significantly higher edema rates compared to the control group at all time points (*p* < 0.05). The positive control group (aspirin) and the Naru‐3 Flavor Pill groups (low, medium, and high doses) all showed significantly lower edema rates compared to the model group at most time points (*p* < 0.05). ELISA results showed that the model group exhibited significantly higher levels of PGE2 and TNF‐α compared to the control group, while the levels of PGE2 and TNF‐α were decreased in Positive and Naru‐3 Flavor Pill groups (low, medium, and high doses) compared to model group (*p* < 0.05; Table 4). Furthermore, serum analysis showed elevated PI3K levels in the model group (732.23 ± 85.86 pg/mL, *p* < 0.05) compared to the control group, with significant elevated of PI3K in the low‐ and middle‐dose groups compared to Model group, and the high‐dose group (737.72 ± 107.16) is essentially similar to the model but higher than the control (*p* < 0.05; Table 4). These results demonstrated that Naru‐3 Flavor Pill has significant anti‐inflammatory effects.

**Table 3:** Comparison of paw edema rates at different time points among groups.

Group	1 h edema rate	2 h edema rate	3 h edema rate	4 h edema rate	5 h edema rate
Control	0.04 ± 0.01	0.04 ± 0.01	0.04 ± 0.01	0.04 ± 0.01	0.02 ± 0.01
Model	0.30 ± 0.12^b^	0.63 ± 0.14^b^	0.71 ± 0.09^b^	0.77 ± 0.09^b^	0.70 ± 0.11^b^
Positive	0.14 ± 0.08^a^	0.21 ± 0.19^a^	0.25 ± 0.14^a^	0.31 ± 0.16^a^	0.23 ± 0.21^a^
Low‐Naru‐3	0.18 ± 0.03^a^	0.46 ± 0.08^a^	0.53 ± 0.09^a^	0.53 ± 0.12^a^	0.55 ± 0.13^a^
Middle‐Naru‐3	0.24 ± 0.07	0.56 ± 0.09	0.62 ± 0.11	0.61 ± 0.12^a^	0.56 ± 0.10^a^
High Naru‐3	0.26 ± 0.07	0.55 ± 0.18	0.57 ± 0.14^a^	0.60 ± 0.13^a^	0.51 ± 0.19^a^

^a^indicates significant difference compared to the model group (*p* < 0.05).

^b^indicates significant difference compared to the control group (*p* < 0.05).

**Table 4:** Comparison of serum levels of inflammatory cytokines among groups.

Group	PGE2 (pg/mL)	IL‐6 (pg/mL)	IL‐1β (pg/mL)	TNF‐α (pg/mL)	PI3K (pg/mL)
Control	48.90 ± 3.33	10.65 ± 3.27	3.15 ± 0.34	32.67 ± 19.37	390.68 ± 82.17
Model	50.77 ± 3.96	13.77 ± 7.03	3.32 ± 0.36	57.79 ± 9.84^b^	732.23 ± 85.86^b^
Positive	43.23 ± 3.61^ab^	7.85 ± 2.86	3.35 ± 0.36	38.43 ± 14.85^a^	887.34 ± 194.76^ab^
Low‐Naru‐3	48.15 ± 3.83	9.89 ± 2.97	3.14 ± 0.34	41.04 ± 20.19^a^	901.77 ± 106.84^ab^
Middle‐Naru‐3	47.40 ± 5.61	5.81 ± 1.21^a^	3.32 ± 0.31	32.51 ± 5.85^a^	876.07 ± 106.92^ab^
High Naru‐3	45.64 ± 4.99^a^	7.28 ± 2.04^a^	3.46 ± 0.24	32.72 ± 6.18^a^	737.72 ± 107.16^b^

^a^indicates significant difference compared to the model group (*p* < 0.05).

^b^indicates significant difference compared to the control group (*p* < 0.05).

### 3.2. Main Serum Components of Naru‐3 Wei Pill

The chemical composition of Naru‐3 Wei Pill components in the blood was determined using HPLC separation coupled with mass spectrometry (positive and negative ion scanning) and analyzed with XCMS software (Figure 1A,B). Through screening with the TCMSP database and searching the literature, we identified 35 compounds with percentage human oral absorption ≥30% and DL ≥0.18, and 328 gene targets for anti‐inflammatory and analgesic compounds. The obtained compounds and targets were imported into Cytoscape 3.9.1 and analyzed in descending order using the cytoHubba algorithm tool (Table 5).

Figure 1HPLC analysis of the main blood‐borne components of Naru‐3 Wei Pill. (A) Cationic mode and (B) anionic mode.

**Figure:**
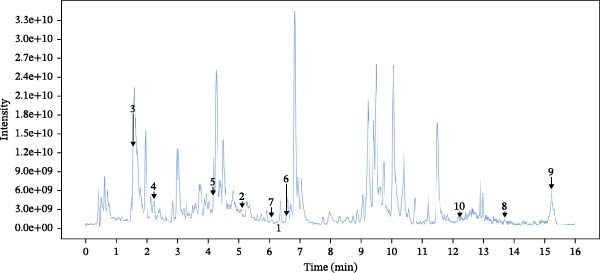
(A)

**Figure:**
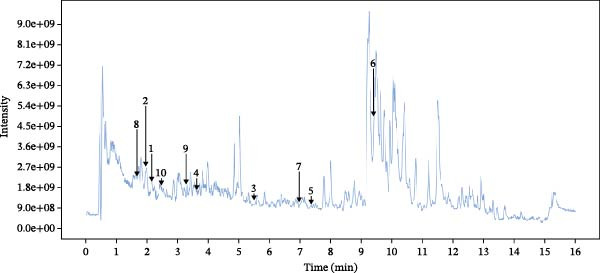
(B)

**Table 5:** Compounds of Naru‐3 Wei Pill in mouse blood with potential anti‐inflammatory and analgesic effects.

Name	Composite score	Formula	ppm	MS2	Percent human oral absorption	DL	Degree
Genistein	0.871582	C_15_H_10_O_5_	1.770563121	269.044764; 209.117661; 207.138695; 59.013984; 92.679428	76.3294	0.218573236	207
Capsaicin	0.788479385	C_18_H_27_NO_3_	1.406672838	328.189654; 58.065234; 121.065197; 283.132659; 137.059506	100	0.732881927	114
Rhein	0.716517615	C_15_H_8_O_6_	1.358993505	239.036941; 283.026087; 268.039017; 195.043957; 211.040779	47.0446	0.202714849	61
Piperlongumine	0.980000769	C_17_H_19_NO_5_	0.872982091	340.11615; 92.657848; 120.154628; 165.76208; 354.147329	100	0.447259924	33
Mangostin		C_24_H_26_O_6_	0.72333996	411.178443; 216.076232; 201.05036; 217.082703; 176.044141	100	0.93655733	32
Nobiletin	0.806888385	C_21_H_22_O_8_	1.081609172	403.137545; 206.054899; 72.080703; 402.263828; 205.096322	100	0.796810742	27
Nonivamide	0.985552385	C_17_H_27_NO_3_	0.527631419	292.192807; 126.056086; 293.194805; 92.676116; 95.122958	100	0.735981951	23
DL‐Laudanosine	0.981442462	C_21_H_27_NO_4_	0.30926395	380.183855; 92.657003; 210.085699; 173.43936; 213.781346	100	0.613559662	19
(R)‐Roemerine	0.961509769	C_18_H_17_NO_2_	10.18636049	280.130479; 92.655298; 248.977108; 158.943958; 295.079632	100	0.365279672	19
Glaucine	0.913968538	C_21_H_25_NO_4_	0.807678565	294.124905; 356.185865; 325.144138; 92.658695; 310.121659	100	0.715165746	15
Bilobol	0.833470154	C_21_H_34_O_2_	0.449967567	336.287623; 180.139431; 95.049035; 112.075512; 86.096653	100	0.749362232	13
Allocryptopine	0.996972923	C_21_H_23_NO_5_	1.409859062	392.143724; 112.075505; 86.096516; 393.147168; 356.18491	95.8831	0.760757532	11
Piperanine	0.975302308	C_17_H_21_NO_3_	0.213146008	310.141665; 92.655311; 267.135457; 311.180423; 235.307452	100	0.512912109	11
Secologanin dimethyl acetal	0.910586462	C_19_H_30_O_11_	4.528544621	435.184265; 243.145295; 434.318496; 92.65603; 138.09101	45.2063	0.784751752	11
1,7‐Dimethyl‐7‐(4‐methyl‐3‐penten‐1‐yl)bicyclo [2.2.1]heptan‐2‐ol	0.906323308	C_15_H_26_O	0.463532332	264.23147; 81.033601; 112.075572; 86.09581; 69.069728	100	0.199651667	11
Petroselinic acid	0.965866154	C_18_H_34_O_2_	0.392390138	57.070251; 69.069637; 95.084897; 83.085622; 97.10124	91.5593	0.752313669	10
Cryptopine	0.873589538	C_21_H_23_NO_5_	0.512617407	370.163614; 369.354038; 147.117327; 161.133011; 109.101057	100	0.786537123	10
Bullatine B	0.950430231	C_24_H_39_NO_6_	0.076397425	438.283686; 420.270156; 388.247007; 154.122475; 58.065372	84.608	0.629377318	9
Urolithin M5	0.875404154	C_13_H_8_O_7_	12.06834059	275.018511; 257.104088; 231.028946; 203.034088; 197.083142	43.5177	0.183743373	9
Isopongaflavone	0.778143385	C_21_H_18_O_4_	0.780001973	267.064812; 149.022775; 352.151544; 201.053427; 86.095872	100	0.555841095	9
Dihydrochelerythrine	0.672324	C_21_H_19_NO_4_	0.241140433	201.053371; 350.136029; 264.157857; 218.080986; 86.096585	100	0.704613821	9
Arctigenin	0.604808	C_21_H_24_O_6_	3.520092037	193.086328; 135.044043; 151.075909; 373.126519; 133.064094	100	0.780621265	9
1‐Methyl‐2‐[(6Z)‐6‐undecen‐1‐yl]‐4(1H)‐quinolinone	1	C_21_H_29_NO	0.04692505	312.228972; 330.240602; 272.255583; 324.593752; 92.656087	100	0.773405722	8
Pellitorine	0.944448077	C_14_H_25_NO	1.385353836	224.200273; 57.069918; 69.069875; 168.137373; 81.032772	100	0.466200487	8
5‐Demethylnobiletin	0.863148846	C_20_H_20_O_8_	1.059841563	411.10719; 410.215452; 216.040403; 201.05359; 131.049516	100	0.731879264	8
Orixanone B	0.808532923	C_17_H_19_NO_4_	1.351927533	302.140652; 232.131794; 138.091099; 112.075474; 86.096488	100	0.352988316	8
4,5‐Dihydropiperlonguminine	0.728480615	C_16_H_21_NO_3_	0.611400757	203.069317; 137.059649; 276.156799; 56.964865; 161.059221	100	0.501451942	8
1,5,15‐Tri‐O‐methylmorindol	0.998424308	C_18_H_16_O_6_	0.198581372	327.088772; 297.039324; 283.098046; 328.089746; 312.023317	90.0689	0.449200836	7
2‐Hydroxy‐3‐methoxy‐12‐methyl‐12h‐[1, 3]dioxolo [4′,5′:4,5]benzo[1,2‐c]‐phenanthridin‐13‐one	0.694732846	C_20_H_15_NO_5_	0.952874684	350.099376; 131.049527; 218.081321; 105.070138; 349.199439	96.5297	0.636969343	7
Leonurine	0.963585385	C_14_H_21_N_3_O_5_	0.568469685	334.139384; 92.657002; 223.241839; 104.197207; 358.4078	64.2074	0.60000449	6
[10]‐Gingerdiol	0.664844846	C_21_H_36_O_4_	0.212838271	307.264355; 72.02144; 135.151754; 71.014293; 217.348145	100	0.64220313	6
(R)‐Higenamine	0.984992462	C_16_H_17_NO_3_	0.389239903	272.123848; 212.078086; 254.11531; 280.789462; 59.326764	66.3757	0.233643918	3
Ginkgolic acid C17:1	0.986312	C_24_H_38_O_3_	1.018309461	329.282669; 373.272909; 330.287662; 374.273381; 92.681193	100	0.59114491	2
(+)‐Usnic acid	0.713150692	C_18_H_16_O_7_	0.675460428	343.079513; 245.010043; 273.004939; 92.679389; 145.014951	70.344	0.400185595	2
Anisodine	0.694219769	C_17_H_21_NO_5_	25.62588738	116.071987; 318.133303; 288.124118; 67.018913; 189.055565	77.302	0.391039121	2

### 3.3. Prediction and Identification of Effective Targets of Naru‐3 Wei Pill

A total of 8795 disease targets of inflammation and pain were collected from the GeneCards, TTD, DisGeNET, and PharmGKB databases, respectively, after searching using the keywords “Inflammation” and “Pain.” The intersection of the predicted 328 gene targets of Naru‐3 Wei Pill and the 8795 disease targets was obtained, resulting in 291 potential targets of anti‐inflammatory and analgesic compounds.

### 3.4. Construction of the Naru‐3 Wei Pills–Active Ingredient–Targets Network and PPI Network

Using Cytoscape 3.9.1, we constructed a network of the interactions of Naru‐3 Wei Pill, its active ingredients, and the predicted targets (Figure 2A). The network contained 327 nodes and 747 edges. All 291 anti‐inflammatory and analgesic targets of Naru‐3 Wei Pill were imported into the STRING database, and a PPI network was constructed (Figure 2B). The network contained 273 nodes (representing proteins) and 2816 edges (representing interactions between proteins). The CytoHubba tool was applied to obtain key targets (degree >62) from this PPI network. The top 10 targets with the largest degree values are shown in Table 6: these were tumor protein p53 (TP53), RAC‐alpha serine/threonine‐protein kinase 1 (AKT1), SRC, epidermal growth factor receptor (EGFR), ESR1, STAT3, mitogen‐activated protein kinase 14 (MAPK14), MAPK1, and NFKB1.

Figure 2Construction of Naru‐3 Wei Pill–active ingredient–target network and PPI network. (A) Naru‐3 Wei Pill–active ingredient–target network. Fluorescent green diamond represents Naru‐3 Wei Pill; green hexagons represent active ingredients of Naru‐3 Wei Pill; yellow squares represent target genes of active ingredients. (B) PPI network of potential targets of Naru‐3 Wei Pill related to its anti‐inflammatory and analgesic effects. Inner circle shows the top 10 hub proteins by degree value.

**Figure:**
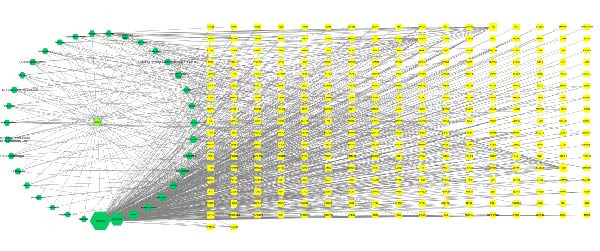
(A)

**Figure:**
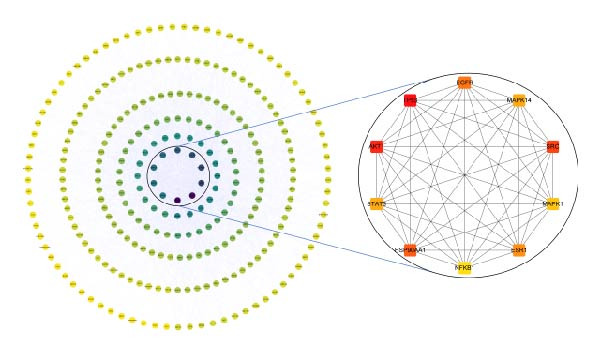
(B)

**Table 6:** Potential targets of Naru‐3 Wei Pill related to its anti‐inflammatory and analgesic effects.

Number	Target protein	Full name in English	Degree
1	TP53	Tumor protein P53	106
2	AKT1	RAC‐alpha serine/threonine‐protein kinase 1	101
3	SRC	Proto‐oncogene tyrosine‐protein kinase Src	83
4	HSP90AA1	Heat shock protein 90 alpha family Class A Member 1	79
5	EGFR	Epidermal growth factor receptor	76
6	ESR1	Estrogen receptor 1	74
7	STAT3	Signal transducer and activator of transcription 3	66
8	MAPK14	Mitogen‐activated protein kinase 14	66
9	MAPK1	Mitogen‐activated protein kinase 1	64
10	NFKB1	Nuclear factor kappa B subunit 1	62

### 3.5. GO Functional Enrichment Analysis and KEGG Pathway Enrichment Analysis

All 291 anti‐inflammatory and analgesic targets of Naru‐3 Wei Pill were imported into the DAVID database; the species was set to human, and GO functional enrichment analysis was carried out (Figure 3A). The targets were enriched in 2699 biological processes, mainly response to drug, second‐messenger‐mediated signaling, vascular processes in circulatory system, adenylate cyclase‐modulating G‐protein‐coupled receptor signaling pathway, peptidyl‐serine modification, regulation of blood vessel diameter, and so on. They were also enriched in 127 cellular components (mainly membrane rafts, membrane regions, synaptic membrane components, postsynaptic membranes, and plasma membrane rafts) and in 269 molecular functions (serine/threonine kinase activity, G‐protein‐coupled peptide receptor activity, peptide receptor activity, nuclear receptor activity, drug binding, catecholamine binding, etc.).

Figure 3GO and KEGG analysis of 291 protein targets of anti‐inflammatory and analgesic active components of Naru‐3 Wei Pill. (A) Go analysis and (B) KEGG enrichment analysis. Length represents the number of target genes, and color represents the level of significance.

**Figure:**
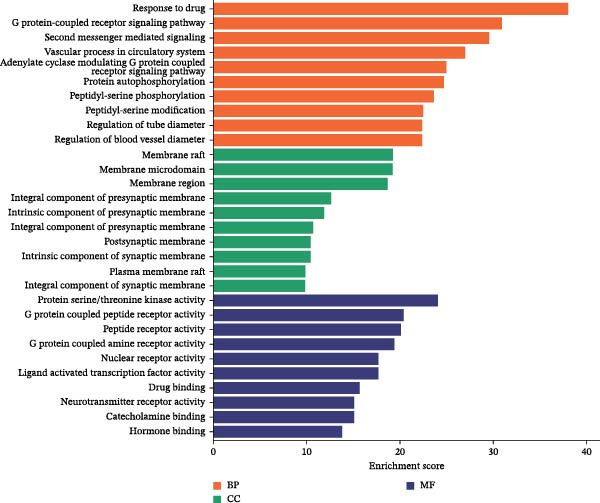
(A)

**Figure:**
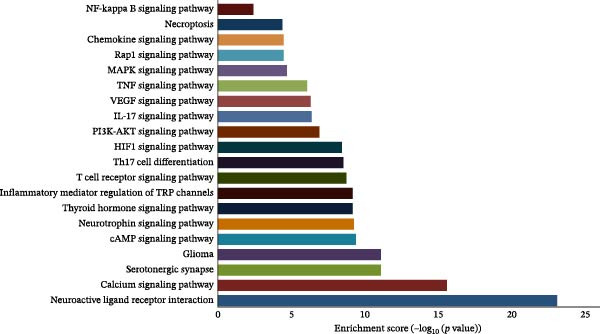
(B)

According to the KEGG analysis, the targets of interest were enriched in 172 pathways. The top 20 pathways (based on adjusted *p* < 0.05; visualized in Figure 3B) were related to anti‐inflammatory and analgesic effects; in particular, calcium signaling pathway, serotonergic synapse, cyclic AMP (cAMP) and neurotrophin signaling pathway, thyroid hormone signaling pathway, T cell receptor pathway, Th17 cell differentiation, HIF‐1 pathway, PI3K/Akt, IL‐17, VEGF, TNF, MAPK, Rap1, chemokine, necroptosis, NF‐κB signaling pathway, and so on. Notably, thyroid hormone signaling regulates immune cell activation and pro‐inflammatory cytokine (e.g., TNF‐α and IL‐6) release, which are core mediators of inflammatory pain; necroptosis drives a pro‐inflammatory cell death program that amplifies tissue inflammation and neural sensitization; Rap1 signaling modulates mechanical pain perception via regulating Piezo2 channel activity in dorsal root ganglion neurons, directly linking to analgesic effects. These pathways are thus functionally associated with the study’s focus on inflammation and pain relief.

### 3.6. Molecular Docking

**Figure 4:**
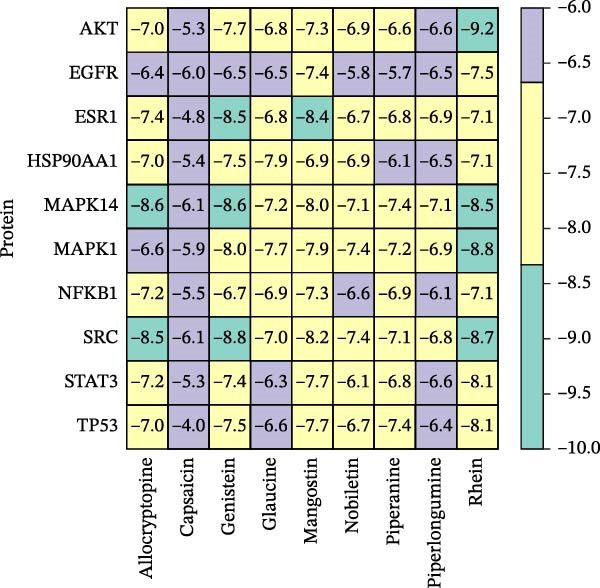
Heat map of binding energies of key targets with core components of Naru‐3 Wei Pill.

Molecular docking was performed using Autodock software to identify potential binding modes and the reliability of the association for 10 key targets (TP53, AKT1, SRC, HSP90AA1, EGFR, ESR1, STAT3, MAPK14, MAPK1, and NFKB1) and nine active compounds (genistein, capsaicin, Rhein, piperlongumine, mangostin, nobiletin, glaucine, allocryptopine, and piperanine) of Naru‐3 Wei Pill. The results were visualized and a heatmap was constructed (Figure 4). If the binding energy between a ligand and receptor is low, the binding stability between them will be high. Here, the binding energies of active ingredients and core proteins were ≤−6 kcal/mol, indicating that the ligands showed good binding to the receptor proteins. The binding energies of AKT1–Rhein (Figure 5A), MAPK1–Rhein (Figure 5B), MAPK14–Rhein (Figure 5C), SRC–Rhein (Figure 5D), ESR1–genistein (Figure 5E), MAPK14–genistein (Figure 5F), SRC–genistein (Figure 5G), ESR1–mangostin (Figure 5H), MAPK14–allocryptopine (Figure 5I), and SRC–allocryptopine (Figure 5J) were all ≤−8 kcal/mol. The lowest binding energy, for AKT1–Rhein, was −9.2 kcal/mol. A molecular docking diagram was produced using PyMOL software.

Figure 5Molecular docking diagram for interactions of main active compounds and potential targets. (A) Rhein–AKT1; (B) Rhein–MAPK1; (C) Rhein–MAPK14; (D) Rhein–SRC; (E) genistein–ESR1; (F) genistein–MAPK14; (G) genistein–SRC; (H) mangostin–ESR1; (I) allocryptopine–MAPK14; (J) allocryptopine–SRC.

**Figure:**
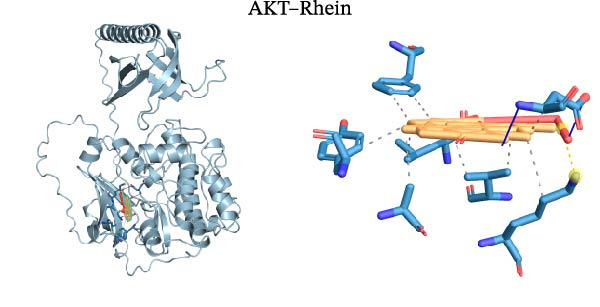
(A)

**Figure:**
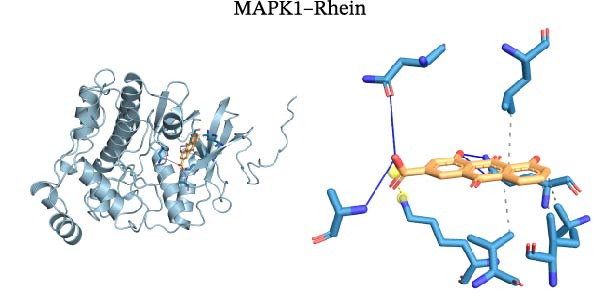
(B)

**Figure:**
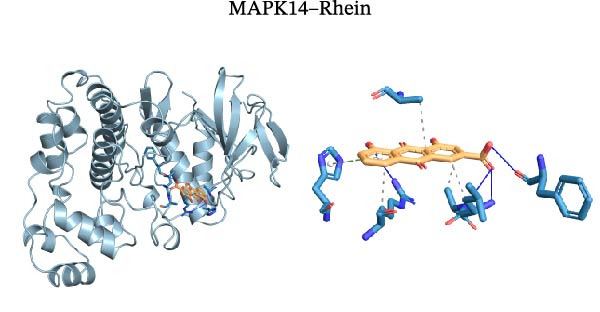
(C)

**Figure:**
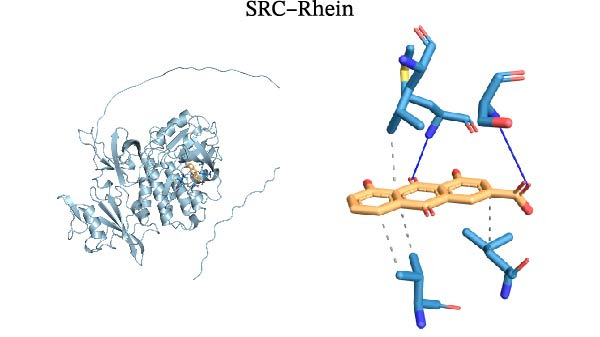
(D)

**Figure:**
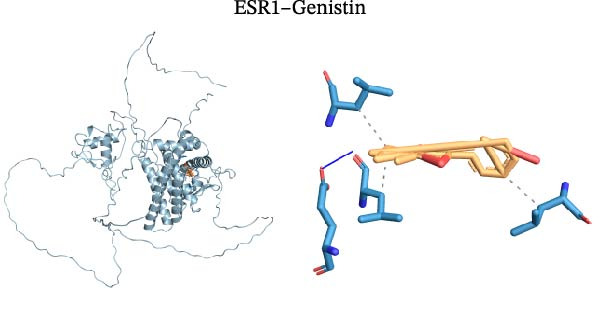
(E)

**Figure:**
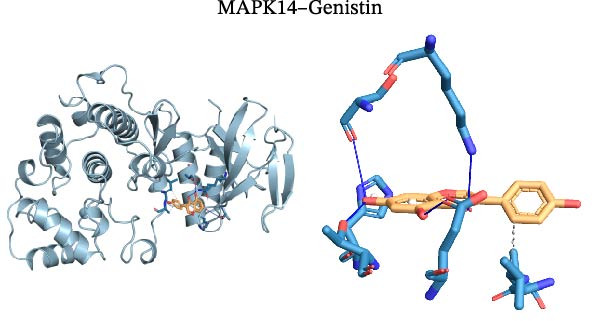
(F)

**Figure:**
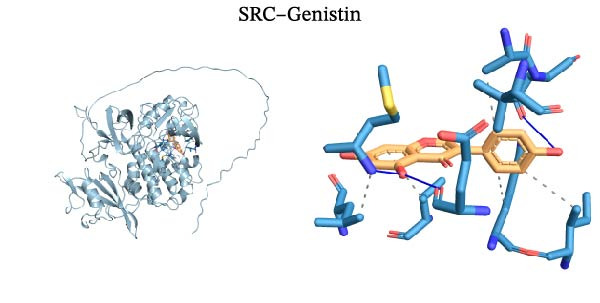
(G)

**Figure:**
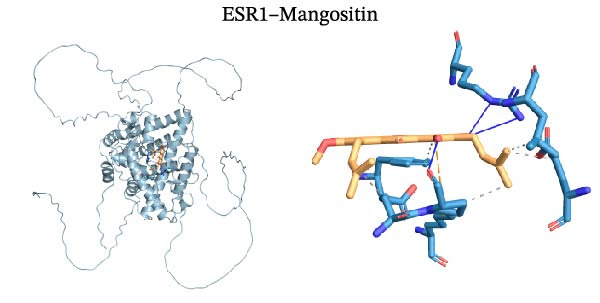
(H)

**Figure:**
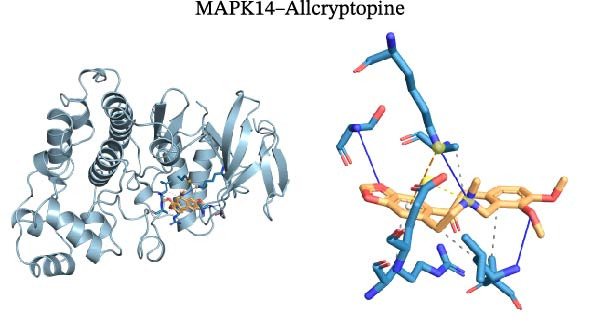
(I)

**Figure:**
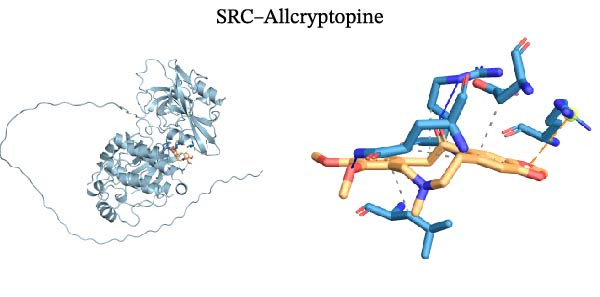
(J)

### 3.7. Root Mean Square Deviation (RMSD) and Root Mean Square Fluctuation (RMSF) of Molecular Dynamics Results

RMSD and RMSF curves are used to reflect fluctuations in protein conformation and amino acid residues, respectively [9]. As shown in Figure 6A, the RMSD results for our molecular dynamics binding simulations showed fluctuations in the early stages; those for SRC–Rhein and SRC–genistein stabilized after 5 ns, whereas those for AKT1–Rhein and MAPK1–Rhein stabilized after 25 ns. The RMSD values in these four cases became stable at 1.0, 1.2, 0.6, and 0.4 Å, respectively. The RMSF analysis revealed higher residue‐level flexibility in SRC complexes compared to AKT1–Rhein and MAPK1–Rhein, with peaks approaching 1.6–1.8 nm (Figure 6B). Although the overall backbone RMSD suggests that the SRC–ligand complexes maintain stable global conformations during simulation, the RMSF analysis reveals enhanced local flexibility of specific amino acid residues, which may reflect region‐specific conformational dynamics relevant to ligand binding and protein function.

Figure 6Molecular dynamics simulations. (A) RMSD values for Rhein–AKT1, Rhein–MAPK1, Rhein–SRC, and genistein–SRC. (B) RMSF values for Rhein–AKT1, Rhein–MAPK1, Rhein–SRC, and genistein–SRC.

**Figure:**
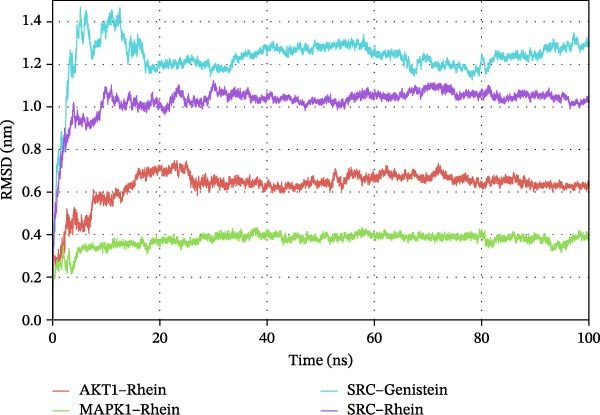
(A)

**Figure:**
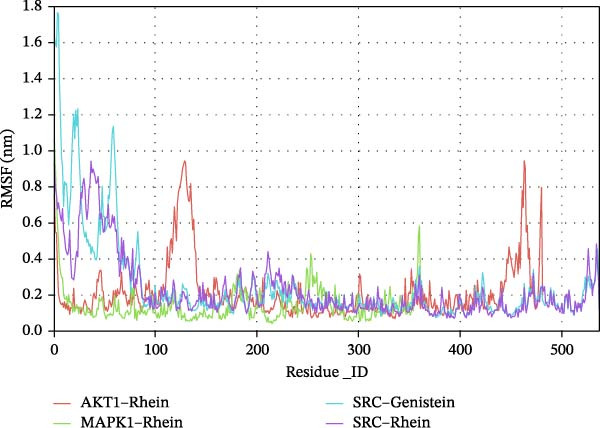
(B)

### 3.8. Naru‐3 Pills Reduced the Expression of mTOR, AKT, and PI3K

Above results have shown that Naru‐3 may regulate the AKT signaling pathway and downregulate the level of PI3K in serum. To further investigate the effect of Naru‐3 on the AKT signaling pathway, we detected the expression of AKT, mTOR, and PI3K in the plantar tissues of rats. The results showed that the expression of AKT, mTOR, and PI3K was significantly upregulated in the model group, while Naru‐3 significantly inhibited the expression of AKT, mTOR, and PI3K both in mRNA and protein level in model + Naru‐3 group (Figure 7). These findings suggest that Naru‐3 may regulate inflammation by modulating the AKT/PI3K signaling pathway.

Figure 7Verification of Naru‐3 Pills on the expression of AKT, mTOR, and PI3K. (A) The mRNA expression of AKT, mTOR, and PI3K in rats paw tissues between control, model, and Naru‐3 high‐dose groups. (B) The protein expression of AKT, mTOR, and PI3K in rats paw tissues between control, model, and Naru‐3 high‐dose groups. Vinculin as a loading control. The results showed that the expression of AKT, mTOR, and PI3K was significantly upregulated in the model group, while Naru‐3 significantly inhibited the expression of AKT, mTOR, and PI3K both in mRNA and protein level in model + Naru‐3 group. Vinculin was used as a loading control to ensure equal protein loading across all experimental groups. As shown, vinculin expression levels remained consistent and showed no obvious differences among all groups.  ^∗∗∗^ *p* < 0.001.

**Figure:**
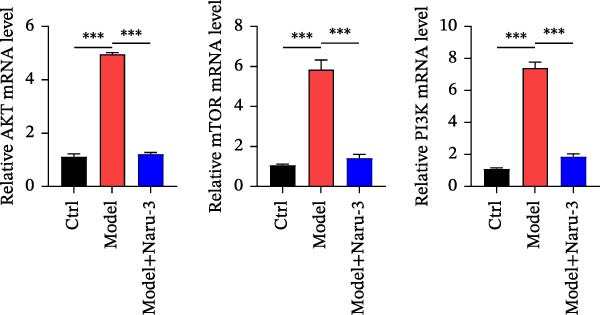
(A)

**Figure:**
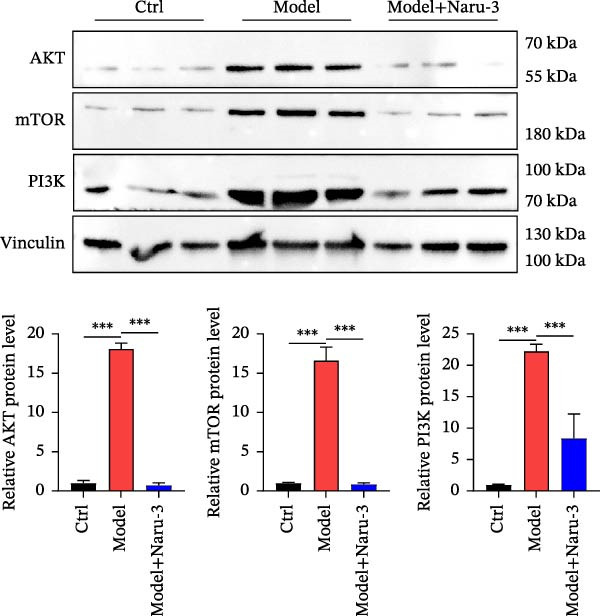
(B)

## 4. Discussion

The Mongolian medicine Naru‐3 has a history of more than 1000 years and has shown significant effects in the treatment of various inflammatory diseases. According to modern pharmacological research, this drug has anti‐inflammatory, analgesic, and antibacterial effects [10]. Naru‐3 Wei Pill is composed of *Terminalia chebula* Retz and *Aconitum kusnezoffii* Reichb. The compatibility ratio with *Piper longum L*. is 10:5:3. To prepare the pill, the components are soaked in deionized water for 3 h, extracted twice with ultrasonic waves (1 h each time), and then thoroughly filtered, followed by drying of the filtrate. The dosage during treatment is 1 g, once per day. Pharmacological studies have shown that *Aconitum carmichaelii* can alleviate pain, *Hedyotis diffusa* can improve the immune system, and pepper can exert the effects of dispelling cold and dampness [11]. The present study investigated the anti‐inflammatory and analgesic effects of Naru‐3 pills using multiple pain models (hot plate, tail flick, acetic acid–induced writhing tests, and paw edema inflammation experiment) and explored their potential mechanisms by measuring serum levels of PI3K, PGE2, IL‐6, IL‐1β, and TNF‐α. The findings provide comprehensive insights into the efficacy and underlying mechanisms of Naru‐3 pills as an anti‐inflammatory and analgesic agent.

Network pharmacology is widely used in the development of new drugs, mainly by establishing a network of relationships among drugs, ingredients, targets, and diseases and then using the network to analyze the mechanism of action of drugs. The results obtained can provide support for drug research and optimization [12]. The present study comprehensively and systematically investigated the anti‐inflammatory and analgesic mechanisms of Naru‐3 Wei Pill through HPLC analysis and network pharmacology methods. First, an interaction network was established, and 35 active compounds were selected. Second, 291 targets were identified by taking the intersection between drug targets and disease targets, and a PPI network was established to analyze the relationships between proteins. Finally, enrichment analysis was conducted, resulting in the identification of 3095 GO terms and 172 related signaling pathways. The stability of the binding between small molecule compounds and target proteins was analyzed, and stable binding was predicted for several compound–protein pairs. Taken together, the network pharmacology, molecular docking, and cell experiment results reveal the anti‐inflammatory and analgesic mechanisms of Naru‐3 Wei Pill.

The HPLC analysis identified genistein, capsaicin, Rhein, piperlongumine, mangostin, nobiletin, glaucine, allocryptopine, and piperanine as potential active components related to anti‐inflammatory and analgesic effects. Genistein inhibits histopathological changes in acute pancreatitis via its antioxidant and anti‐inflammatory activity [13]. Animal experiments have shown that capsaicin supplementation can increase the expression of IL‐10 and inhibit that of IL‐1 β and IL‐8, thereby alleviating many inflammation‐related diseases (such as gastritis). In clinic settings, capsaicin is often used to relieve chronic pain or neuropathic pain caused by diseases including rheumatoid arthritis and postherpetic neuralgia [14, 15]. Rhubarb is a bioactive ingredient that has an inhibitory effect on the expression of pro‐inflammatory factors and can also block the secretion of inflammatory mediators; thus, it exhibits high anti‐inflammatory activity. In addition, this medication is considered safe and causes minimal damage to the gastrointestinal tract [16]. Nobiletin has pharmacological activities including anti‐carcinogenic, anti‐inflammatory, and anti‐obesity functions [17–19], whereas piperanine has anti‐inflammatory, analgesic, antioxidant, antibacterial, anticancer, and antiepileptic activities [20].

Based on the PPI network constructed in this study, TP53, AKT1, SRC, HSP90AA1, EGFR, ESR1, STAT3, MAPK14, MAPK1, and NFKB1 were found to be most closely related to the anti‐inflammatory and analgesic effects of Naru‐3 Wei Pill and were identified as essential target proteins. p53 is a key factor in blocking inflammatory responses via many pathways [21] and can inhibit major inflammatory transcription factor NF‐κB [22]. AKT1 is of great significance in regulating cell metabolism, growth, apoptosis, and survival and has a carcinogenic effect [23]. SRC, a protein tyrosine kinase, can regulate osteoclast function through various signaling pathways and promote the aggregation of phosphatidylinositol 3‐kinase. It can also promote osteoclast proliferation by activating cytochrome c oxidase and activates the Yes‐associated protein 1–Notch signaling pathway to mediate IL‐6 signaling and reduce inflammation [24].

Heat shock proteins (HSPs) have important roles in maintaining cellular homeostasis and provide support for the body to cope with adverse external stimuli. These proteins can inhibit inflammation and increase cellular stress levels and thus also have a defensive role. For example, HSP90α has been postulated to signal fatigue in chronic inflammation [25]. It is reported that when EGFR is phosphorylated, it can activate downstream PI3K/AKT, NF‐κB, and other pathways that are involved in the inflammatory response [26]. ESR1 is an estrogen receptor, and its levels or activity may be affected by chronic inflammation, leading to aberrant tumor cell signaling [27]. STAT3 has a key function in inducing and maintaining the inflammatory microenvironment. It was initially regarded as an acute phase response factor activated after IL‐6 stimulation, and its activation in macrophages and neutrophils has proved to be essential for preventing chronic inflammation in mice [28]. MAPK14 is a key factor in alleviating the inhibition and control of inflammation by stress‐signal–induced autophagy. It can effectively inhibit the activity of Ulk1 kinase and prevent its interaction with the Atg13 complex, as well as alleviating the inhibition of inflammation by autophagy to achieve a comprehensive immune response [29]. MAPK1 is induced by phosphorylation to activate the expression of transcription factors, including NF‐κB and activated protein‐1 (AP‐1), and plays an important part in inflammatory response [30]. NFKB1 is an essential regulator of NF‐κB activity in vivo and a suppressor of inflammation, aging, and cancer [31].

According to the GO enrichment results, the anti‐inflammatory and analgesic effects of Naru‐3 Wei Pill are mainly associated with drug reactions, G‐protein‐coupled receptor signaling pathway, second messenger‐mediated signal transduction, vascular processes in the circulatory system, and so on, whereas the KEGG analysis indicated essential associations with the cAMP signaling pathway, Th17 cell differentiation, IL‐17 signaling pathway, HIF‐1, PI3K/Akt, TNF, MAPK, and NF‐κB signaling pathway. cAMP can cause hyperactivity and hyperalgesia of sensory neurons [32]; hyperalgesia in long‐term opioid addicts during withdrawal is also caused by elevated cAMP levels [33]. The T cell receptor signaling pathway is an important pathway by which T cells recognize antigens and signaling factors and produce antigen‐specific immunity; it has been implicated in the therapeutic effect of Shaogan Fuzi Decoction on rheumatoid arthritis [34]. IL‐17 is closely related to the pathogenesis of several inflammatory diseases; for instance, targeting IL‐17 and Th17 cells in chronic inflammation has yielded positive results in treatment of psoriasis and ankylosing spondylitis in both mouse models of disease and clinical settings [35]. Hypoxia and inflammation have key roles in the pathological processes of many diseases and have an interactive relationship. HIF is activated under normoxic conditions; therefore, inflammation may occur before tissue hypoxia [36]. The PI3K/Akt axis promotes activation of innate immune cells, leading to inflammation [37]. Abnormal transmission of this pathway in adipose tissue can lead to failure to relieve inflammation in obese patients, which is closely related to their symptoms [38]. TNF is a pro‐inflammatory cytokine. Sharp increases in its levels can lead to disease, and studies have found that it exerts inflammatory and analgesic effects by regulating the TNF signaling pathway [39]. MAPK is related to cell proliferation, differentiation, apoptosis, and inflammation, and the MAPK pathway, an important inflammatory signaling pathway, is closely related to inflammation and cancer [40]. The NF‐κB signaling pathway is a key pathway for the treatment of inflammation. Studies have found that inflammatory reactions such as pain, fever, and edema are related to the abnormal expression of inflammatory factors, and diseases involving such processes can be treated by inhibiting the activation of this pathway [41].

Molecular docking technology is mainly used to evaluate the binding strength between compounds and target proteins and is widely used in pharmacological research [42]. It can also be used to analyze the binding stability of ligands and receptors [43]. In the present study, we identified four complexes (SRC–Rhein, SRC–genistein, AKT1–Rhein, and MAPK1–Rhein) as having stable conformation and structure using molecular dynamics simulations. In traditional Mongolian medicine (TMM), “blood stasis (Badgan‐induced stagnation)” is closely associated with inflammatory pain and tissue congestion. Our data show that SRC mediates the phosphorylation of downstream pro‐inflammatory kinases (e.g., AKT1 and MAPK14), which aligns with TMM’s emphasis on “resolving stasis to alleviate pain.” Historical TMM records indicate that the formula’s key ingredients (e.g., *Aconitum kusnezoffii* and *Lonicera japonica*) are traditionally used to dispel stagnant Qi and blood; our findings suggest SRC inhibition may be a modern pharmacological correlate of this traditional effect. Furthermore, TMM posits that “heat‐toxin (Shira excess)” drives inflammatory responses, manifesting as redness, swelling, and pain. AKT1 and MAPK14 are critical regulators of pro‐inflammatory cytokine production (TNF‐α and IL‐6) and NF‐κB activation—molecular events that correspond to “heat‐toxin accumulation” in TMM. Our results demonstrate that the formula downregulates AKT1/MAPK14 signaling, which echoes the traditional use of the formula to “clear heat‐toxin” and suppress excessive inflammatory responses. We further referenced TMM pharmacopeia records confirming the formula’s efficacy in treating inflammatory conditions linked to “Shira‐Badgan imbalance,” reinforcing the target‐mediated mechanism underlying its traditional application.

Furthermore, we demonstrated that Naru‐3 significantly inhibited the expression of key components of the AKT/PI3K pathway, including AKT, mTOR, and PI3K, both at the mRNA and protein levels in the plantar tissues of rats using RT‐qPCR and western blot. The AKT/PI3K pathway is known to play a crucial role in regulating various cellular processes, including cell survival, proliferation, and inflammation [44]. Furthermore, we noted that the AKT/PI3K pathway is a key signaling hub that modulates inflammation by regulating the production of pro‐inflammatory cytokines (e.g., TNF‐α, IL‐6, and IL‐1β), promoting inflammatory cell infiltration, and activating downstream pro‐inflammatory transcription factors. The upregulation of this pathway has been implicated in the pathogenesis of several inflammatory diseases. By inhibiting the expression of AKT, mTOR, and PI3K, Naru‐3 may disrupt the downstream signaling cascade, thereby attenuating the inflammatory response. This is consistent with our findings that Naru‐3 significantly reduced paw edema and the levels of inflammatory cytokines such as PGE2, IL‐6, IL‐1β, and TNF‐α in the serum, as reported in the previous sections. While no obvious adverse effects (e.g., changes in animal behavior, body weight, or organ morphology) were observed in our in vivo experiments during the study period. To address this gap, we plan to conduct dedicated safety and toxicity evaluations in our future research, including: (1) acute toxicity studies to determine the median lethal dose (LD_50_) and dose‐response relationships; (2) subchronic/chronic toxicity assessments to monitor long‐term effects on major organs (e.g., liver, kidney, and heart) via histopathological and biochemical analyses; (3) investigations into potential toxic mechanisms (e.g., alkaloid metabolism in *Aconitum kusnezoffii*).

## 5. Conclusions

In summary, we applied animal models, serum pharmacochemistry, network pharmacology, and molecular dynamics to uncover the anti‐inflammatory and analgesic mechanisms of Naru‐3 Wei Pill, identified 35 active components targeting key pathways and core proteins like AKT1 and SRC. Molecular interactions were confirmed, validating the Pill’s therapeutic potential. PCR and western blot assays confirmed that Naru‐3 may regulate inflammation by modulating the AKT/PI3K signaling pathway. Future studies should focus on further elucidating the molecular mechanisms underlying the interaction between Naru‐3 and the AKT/PI3K pathway, as well as exploring the potential clinical applications of Naru‐3 in treating inflammatory diseases. Further research into the long‐term effects and optimal dosing of Naru‐3 is essential for clarifying its therapeutic potential.

## Author Contributions

**Zhiqiang Han:** conceptualization, supervision, funding acquisition, writing – review and editing. **Aidi Zhang and Husile Kang:** methodology, data curation, formal analysis, writing – original draft. **Qigeqin, Baolechaolu, Wulanqiqige, and Huan Wang:** methodology, visualization, data curation, writing – review and editing. **Yanhua Xu, HaSi, Anda, and Lan Xue:** data curation, writing – review and editing.

## Funding

This study was supported by the Science and Technology Program of Inner Mongolia Autonomous Region (Grant 2022YFSH0114).

## Disclosure

All authors have read and agreed to the published version of the manuscript.

## Ethics Statement

The animal experimental protocols were approved by the Affiliated Hospital of Inner Mongolia Minzu University. The certificate number is NM‐LL‐2023‐03‐15‐04.

## Conflicts of Interest

The authors declare no conflicts of interest.

## Data Availability

The datasets generated and/or analyzed during the current study are available from the corresponding author upon reasonable request.

## References

[1] Board, Mongolian Medicine Editorial Committee , Chinese Medical Encyclopedia, Mongolian Medicine, 1992, Shanghai Science and Technology Press.

[2] National Pharmacopoeia Committee , Drug Standard of the Ministry of Health of the People’s Republic of China: Mongolian Medicine, 1998, Inner Mongolia Science and Technology Press.

[3] Zhao H. , Duan S. , and Shi Y. , et al.Naru-3 Inhibits Inflammation, Synovial Hyperplasia, and Neovascularization in Collagen-Induced Arthritis in Rats, Journal of Ethnopharmacology. (2023) 311, 10.1016/j.jep.2023.116350, 116350. 37019159

[4] Guo J. , Xue J. , He Z. , Jia H. , and Yang X. , The Mechanism by Which Naru 3 Pill Protects Against Intervertebral Disc Cartilage Endplate Degeneration Based on Network Pharmacology and Experimental Verification, Journal of Orthopaedic Surgery and Research. (2023) 18, no. 1, 10.1186/s13018-023-04014-x. PMC10388481 37525208

[5] Baiyila B. , He B. , He G. , and Long T. , Anti-Inflammatory Effect of Mongolian Drug Naru-3 on Traumatic Spinal Cord Injury and Its Mechanism of Action, Journal of International Medical Research. (2018) 46, no. 6, 2346–2358, 10.1177/0300060518760157, 2-s2.0-85049020000. 29614905 PMC6023071

[6] Zhou F. T. , Zong Y. , and Li Y. B. , et al.Mechanism of Mongolian Drug Naru-3 in Initiation of Neuroinflammation of Neuropathic Pain From MMP9/IL-1Beta Signaling Pathway, Zhongguo Zhong Yao Za Zhi. (2023) 48, no. 15, 4173–4186, 10.19540/j.cnki.cjcmm.20230216.401. 37802786

[7] Yu G. , Zhang Y. , and Ren W. , et al.Network Pharmacology-Based Identification of Key Pharmacological Pathways of Yin-Huang-Qing-Fei Capsule Acting on Chronic Bronchitis, International Journal of Chronic Obstructive Pulmonary Disease. (2017) 12, 85–94, 10.2147/COPD.S121079, 2-s2.0-85007553378. 28053519 PMC5191847

[8] Zhu X. , Yao Q. , and Yang P. , et al.Multi-Omics Approaches for in-Depth Understanding of Therapeutic Mechanism for Traditional Chinese Medicine, Frontiers in Pharmacology. (2022) 13, 10.3389/fphar.2022.1031051, 1031051. 36506559 PMC9732109

[9] Ye J. , Li L. , and Hu Z. , Exploring the Molecular Mechanism of Action of Yinchen Wuling Powder for the Treatment of Hyperlipidemia, Using Network Pharmacology, Molecular Docking, and Molecular Dynamics Simulation, BioMed Research International. (2021) 2021, no. 1, 10.1155/2021/9965906, 9965906. 34746316 PMC8568510

[10] Li S. Y. and Bagenna B. , The Advance in Studies on Mongolian Drug Naru-3, Journal of Inner Mongolia University for Nationalities. (2008) 23, 92–94.

[11] Yu J. W. , Yuan H. W. , Bao L. D. , and Si L. G. , Interaction Between Piperine and Genes Associated With Sciatica and Its Mechanism Based on Molecular Docking Technology and Network Pharmacology, Molecular Diversity. (2021) 25, no. 1, 233–248, 10.1007/s11030-020-10055-9. 32130644 PMC7870775

[12] Zhang H. , Yao S. , and Zhang Z. , et al.Network Pharmacology and Experimental Validation to Reveal the Pharmacological Mechanisms of Liuwei Dihuang Decoction Against Intervertebral Disc Degeneration, Drug Design, Development and Therapy. (2021) 15, 4911–4924, 10.2147/DDDT.S338439. 34880601 PMC8648103

[13] Siriviriyakul P. , Sriko J. , Somanawat K. , Chayanupatkul M. , Klaikeaw N. , and Werawatganon D. , Genistein Attenuated Oxidative Stress, Inflammation, and Apoptosis in L-Arginine Induced Acute Pancreatitis in Mice, BMC Complementary Medicine and Therapies. (2022) 22, no. 1, 10.1186/s12906-022-03689-9. PMC9351145 35927726

[14] Lu M. , Chen C. , and Lan Y. , et al.Capsaicin-the Major Bioactive Ingredient of Chili Peppers: Bio-Efficacy and Delivery Systems, Food & Function. (2020) 11, no. 4, 2848–2860, 10.1039/D0FO00351D. 32246759

[15] Srinivasan K. , Biological Activities of Red Pepper (*Capsicum annuum*) and Its Pungent Principle Capsaicin: A Review, Critical Reviews in Food Science and Nutrition. (2016) 56, no. 9, 1488–1500, 10.1080/10408398.2013.772090, 2-s2.0-84975109822. 25675368

[16] Wang H. , Yang D. , Li L. , Yang S. , Du G. , and Lu Y. , Anti-Inflammatory Effects and Mechanisms of Rhein, an Anthraquinone Compound, and Its Applications in Treating Arthritis: A Review, Natural Products and Bioprospecting. (2020) 10, no. 6, 445–452, 10.1007/s13659-020-00272-y. 33128198 PMC7648819

[17] Moazamiyanfar R. , Rezaei S. , and AliAshrafzadeh H. , et al.Nobiletin in Cancer Therapy; Mechanisms and Therapy Perspectives, Current Pharmaceutical Design. (2023) 29, no. 22, 1713–1728, 10.2174/1381612829666230426115424. 37185325

[18] Cui Y. , Wu J. , and Jung S. C. , et al.Anti-Neuroinflammatory Activity of Nobiletin on Suppression of Microglial Activation, Biological and Pharmaceutical Bulletin. (2010) 33, no. 11, 1814–1821, 10.1248/bpb.33.1814, 2-s2.0-78249239462. 21048305

[19] Nakajima A. and Ohizumi Y. , Potential Benefits of Nobiletin, A Citrus Flavonoid, Against Alzheimer’s Disease and Parkinson’s Disease, International Journal of Molecular Sciences. (2019) 20, no. 14, 10.3390/ijms20143380, 2-s2.0-85069766664, 3380. 31295812 PMC6678479

[20] Heqian x. and Jia Gu , Piperine Can Improve Pancreatic β-Cell Apoptosis in Diabetic Mice Induced by High-Fat Diet and Streptozotocin, *Abstracts of the 7th Cross Strait and Hong Kong and Macao Nutrition Science Conference*, 2022, Chinese Nutrition Society, Chinese Student Nutrition and Health Promotion Association.

[21] Shi D. and Jiang P. , A Different Facet of p53 Function: Regulation of Immunity and Inflammation During Tumor Development, Frontiers in Cell and Developmental Biology. (2021) 9, 10.3389/fcell.2021.762651, 762651. 34733856 PMC8558413

[22] Barabutis N. , Schally A. V. , and Siejka A. , P53, GHRH, Inflammation and Cancer, EBioMedicine. (2018) 37, 557–562, 10.1016/j.ebiom.2018.10.034, 2-s2.0-85055032144. 30344124 PMC6284454

[23] Chen Y. J. , Wu C. S. , and Shieh J. J. , et al.Baicalein Triggers Mitochondria-Mediated Apoptosis and Enhances the Antileukemic Effect of Vincristine in Childhood Acute Lymphoblastic Leukemia CCRF-CEM Cells, Evidence-Based Complementary and Alternative Medicine. (2013) 2013, 10.1155/2013/124747, 2-s2.0-84874543473, 124747. 23476680 PMC3580913

[24] Fang Q. , Zhou C. , and Nandakumar K. S. , Molecular and Cellular Pathways Contributing to Joint Damage in Rheumatoid Arthritis, Mediators of Inflammation. (2020) 2020, 10.1155/2020/3830212, 3830212. 32256192 PMC7103059

[25] Grimstad T. , Kvivik I. , Kvaloy J. T. , Aabakken L. , and Omdal R. , Heat-Shock Protein 90alpha in Plasma Reflects Severity of Fatigue in Patients With Crohn’s Disease, Innate Immunity. (2020) 26, no. 2, 146–151, 10.1177/1753425919879988, 2-s2.0-85074103255. 31601148 PMC7016405

[26] Elson D. A. , Ryan H. E. , Snow J. W. , Johnson R. , and Arbeit J. M. , Coordinate Up-Regulation of Hypoxia Inducible Factor (HIF)-1alpha and HIF-1 Target Genes During Multi-Stage Epidermal Carcinogenesis and Wound Healing, Cancer Research. (2000) 60, no. 21, 6189–6195. 11085544

[27] Murray J. I. , West N. R. , Murphy L. C. , and Watson P. H. , Intratumoural Inflammation and Endocrine Resistance in Breast Cancer, Endocrine-Related Cancer. (2015) 22, no. 1, R51–R67, 10.1530/ERC-14-0096, 2-s2.0-84923591240. 25404688

[28] Fan Y. , Mao R. , and Yang J. , NF-KappaB and STAT3 Signaling Pathways Collaboratively Link Inflammation to Cancer, Protein & Cell. (2013) 4, no. 3, 176–185, 10.1007/s13238-013-2084-3, 2-s2.0-84874893354. 23483479 PMC4875500

[29] She H. , He Y. , Zhao Y. , and Mao Z. , Release the Autophage Brake on Inflammation: The MAPK*14*/p38Alpha-ULK1 Pedal, Autophagy. (2018) 14, 1097–1098, 10.1080/15548627.2018.1446626, 2-s2.0-85046805503. 29749797 PMC6103409

[30] Huang S. P. , Guan X. , and Kai G. Y. , et al.Broussonin E Suppresses LPS-Induced Inflammatory Response in Macrophages via Inhibiting MAPK Pathway and Enhancing JAK2-STAT3 Pathway, Chinese Journal of Natural Medicines. (2019) 17, no. 5, 372–380, 10.1016/S1875-5364(19)30043-3, 2-s2.0-85066405437. 31171272

[31] Cartwright T. , Perkins N. D. , and Wilson C. L. , NFKB1: A Suppressor of Inflammation, Ageing and Cancer, The FEBS Journal. (2016) 283, no. 10, 1812–1822, 10.1111/febs.13627, 2-s2.0-85014631893. 26663363

[32] Song X. J. , Wang Z. B. , Gan Q. , and Walters E. T. , CAMP and cGMP Contribute to Sensory Neuron Hyperexcitability and Hyperalgesia in Rats With Dorsal Root Ganglia Compression, Journal of Neurophysiology. (2006) 95, no. 1, 479–492, 10.1152/jn.00503.2005, 2-s2.0-33644801168. 16120663

[33] Bie B. , Peng Y. , Zhang Y. , and Pan Z. Z. , CAMP-Mediated Mechanisms for Pain Sensitization During Opioid Withdrawal, The Journal of Neuroscience. (2005) 25, no. 15, 3824–3832, 10.1523/JNEUROSCI.5010-04.2005, 2-s2.0-17644421106. 15829634 PMC6724939

[34] Shi L. , Zhao Y. , and Feng C. , et al.Therapeutic Effects of Shaogan Fuzi Decoction in Rheumatoid Arthritis: Network Pharmacology and Experimental Validation, Frontiers in Pharmacology. (2022) 13, 10.3389/fphar.2022.967164, 967164. 36059943 PMC9428562

[35] Miossec P. and Kolls J. K. , Targeting IL-17 and TH17 Cells in Chronic Inflammation, Nature Reviews Drug Discovery. (2012) 11, no. 10, 763–776, 10.1038/nrd3794, 2-s2.0-84866934694. 23023676

[36] Eltzschig H. K. and Carmeliet P. , Hypoxia and Inflammation, New England Journal of Medicine. (2011) 364, no. 7, 656–665, 10.1056/NEJMra0910283, 2-s2.0-79951829343. 21323543 PMC3930928

[37] Hawkins P. T. and Stephens L. R. , PI3K Signalling in Inflammation, Biochimica et Biophysica Acta (BBA). (2015) 1851, no. 6, 882–897, 10.1016/j.bbalip.2014.12.006, 2-s2.0-84935923752. 25514767

[38] Acosta-Martinez M. and Cabail M. Z. , The PI3K/Akt Pathway in Meta-Inflammation, International Journal of Molecular Sciences. (2022) 23, no. 23, 10.3390/ijms232315330, 15330. 36499659 PMC9740745

[39] Conaghan P. G. , Cook A. D. , Hamilton J. A. , and Tak P. P. , Therapeutic Options for Targeting Inflammatory Osteoarthritis Pain, Nature Reviews Rheumatology. (2019) 15, no. 6, 355–363, 10.1038/s41584-019-0221-y, 2-s2.0-85065523351. 31068673

[40] Yong H. Y. , Koh M. S. , and Moon A. , The p38 MAPK Inhibitors for the Treatment of Inflammatory Diseases and Cancer, Expert Opinion on Investigational Drugs. (2009) 18, no. 12, 1893–1905, 10.1517/13543780903321490, 2-s2.0-72249120832. 19852565

[41] Xiang H. C. , Lin L. X. , and Hu X. F. , et al.AMPK Activation Attenuates Inflammatory Pain Through Inhibiting NF-KappaB Activation and IL-1beta Expression, Journal of Neuroinflammation. (2019) 16, no. 1, 10.1186/s12974-019-1411-x, 2-s2.0-85061493254. PMC6373126 30755236

[42] Xia Q. D. , Xun Y. , and Lu J. L. , et al.Network Pharmacology and Molecular Docking Analyses on Lianhua Qingwen Capsule Indicate Akt1 Is a Potential Target to Treat and Prevent COVID-19, Cell Proliferation. (2020) 53, no. 12, 10.1111/cpr.12949, e12949. 33140889 PMC7705900

[43] Rehman H. M. , Sajjad M. , and Ali M. A. , et al.Identification of RdRp Inhibitors Against SARS-CoV-2 Through E-Pharmacophore-Based Virtual Screening, Molecular Docking and MD Simulations Approaches, International Journal of Biological Macromolecules. (2023) 237, 10.1016/j.ijbiomac.2023.124169, 124169. 36990409 PMC10043960

[44] Klein-Goldberg A. , Voloshin T. , and Zemer Tov E. , et al.Role of the PI3K/AKT Signaling Pathway in the Cellular Response to Tumor Treating Fields (TTFields), Cell Death & Disease. (2025) 16, no. 1, 10.1038/s41419-025-07546-8. PMC11950169 40148314

